# Primary Stability Optimization by Using Fixtures with Different Thread Depth According To Bone Density: A Clinical Prospective Study on Early Loaded Implants

**DOI:** 10.3390/ma12152398

**Published:** 2019-07-27

**Authors:** Christian Makary, Abdallah Menhall, Carole Zammarie, Teresa Lombardi, Seung Yeup Lee, Claudio Stacchi, Kwang Bum Park

**Affiliations:** 1 Department of Oral Surgery, St Joseph University, Beirut 17-5208, Lebanon; 2 Private practice, Beirut 17-5208, Lebanon; 3 Private Practice, 87011 Cassano allo Ionio, Italy; 4 GyeongSan Mir Dental Hospital, Gyeongsan 41934, Korea; 5 Department of Medical, Surgical and Health Sciences, University of Trieste, 34127 Trieste, Italy; 6 Daegu Mir Dental Hospital, Jung-Gu, Daegu 41934, Korea

**Keywords:** dental implants, osseointegration, bone density, torque, early loading, implant macrodesign

## Abstract

**Background:** Macro- and micro-geometry are among the factors influencing implant stability and potentially determining loading protocol. The purpose of this study was to test a protocol for early loading by controlling implant stability with the selection of fixtures with different thread depth according to the bone density of the implant site. **Materials and Methods:** Patients needing implant therapy for fixed prosthetic rehabilitation were treated by inserting fixtures with four different thread diameters, selected based on clinical assessment of bone quality at placement (D1, D2, D3, and D4, according to Misch classification). Final insertion torque (IT) and implant stability quotient (ISQ) were recorded at baseline and ISQ measurements repeated after one, two, three, and four weeks. At the three-week measurement (four weeks after implant replacement), implants with ISQ > 70 Ncm were functionally loaded with provisional restorations. Marginal bone level was radiographically measured 12 months after implant insertion. **Results:** Fourteen patients were treated with the insertion of forty implants: Among them, 39 implants showing ISQ > 70 after 3 weeks of healing were loaded with provisional restoration. Mean IT value was 82.3 ± 33.2 Ncm and varied between the four different types of bone (107.2 ± 35.6 Ncm, 74.7 ± 14.0 Ncm, 76.5 ± 31.1 Ncm, and 55.2 ± 22.6 Ncm in D1, D2, D3, and D4 bone, respectively). Results showed significant differences except between D2 and D3 bone types. Mean ISQ at baseline was 79.3 ± 4.3 and values in D1, D2, D3, and D4 bone were 81.9 ± 2.0, 81.1 ± 1.0, 78.3 ± 3.7, and 73.2 ± 4.9, respectively. Results showed significant differences except between D1 and D2 bone types. IT and ISQ showed a significant positive correlation when analyzing the entire sample (p = 0.0002) and D4 bone type (p = 0.0008). The correlation between IT and ISQ was not significant when considering D1, D2, and D3 types (p = 0.28; p = 0.31; p = 0.16, respectively). ISQ values showed a slight drop at three weeks for D1, D2, and D3 bone while remaining almost unchanged in D4 bone. At 12-month follow-up, all implants (39 early loading, 1 conventional loading) had satisfactory function, showing an average marginal bone loss of 0.12 ± 0.12 mm, when compared to baseline levels. Conclusion: Matching implant macro-geometry to bone density can lead to adequate implant stability both in hard and soft bone. High primary stability and limited implant stability loss during the first month of healing could allow the application of early loading protocols with predictable clinical outcomes.

## 1. Introduction

Oral implantology is a reliable and predictable technique for edentulous space rehabilitation [1]. Reaching this goal depends on the ability of dental implants to achieve osseointegration [2], and an adequate implant stability is a fundamental prerequisite to avoid detrimental micromovements which could lead to a fibrous encapsulation of the fixture during the healing period, resulting in implant failure [3,4]. This process starts with an initial stage of primary mechanical stabilization followed by an ensuing biological phase that provides secondary implant stability [5].

Secondary stability is a dynamic physiologic healing process mainly affected by implant surface microtopography [6], while implant primary mechanical stability is a surgical outcome influenced by various factors, including bone density, implant macro- and micro-geometry, and implant bed preparation technique [7].

Primary stability can be determined upon implant placement by measuring insertion torque (IT) values or by means of resonance frequency analysis measurement (Implant Stability Quotient-ISQ) [8]. IT and ISQ can be influenced by many clinical and implant-related parameters. Bone density was shown to proportionally affect both IT and ISQ values: The denser the bone, the higher both values were [9,10]. Nevertheless, it is always difficult to attain optimal implant stability in all bone types. High IT and ISQ values are easily achieved in hard bone, while low values are often observed in soft bone conditions [11]. Both of these occurrences may negatively impact on the healing outcomes, since very high IT values may excessively compress the cortical bone, leading to early marginal bone loss [12,13], whereas low IT values could impede early healing and bone-to-implant interface quality [10]. Thus, it is always important to control implant primary stability upon placement in order to predictably achieve osseointegration [14]. Although bone quality is a factor that cannot be controlled, many authors have focused on different means to control implant primary stability. Implant site preparation technique and choice of implant body geometry are usually helpful to control final implant seating outcome [7]. Undersized implant site preparation technique was shown to ensure higher IT values in soft bone without altering bone healing around dental implants [15,16]. Nevertheless, histological studies showed that implant placed in excessively undersized osteotomies in hard bone induced microcracks in the cortical layer, leading to necrosis and remodeling that may compromise implant stability at early healing stages [17]. Moreover, the use of cylindrical implants in hard bone and tapered implants in soft bone are recognized techniques that help to control final IT values. In a cadaver study, a comparison between different implant designs concluded that cylindrical implants achieved good stability in hard and intermediate bone, but failed to achieve good stability in soft bone while tapered implants appeared to reach higher primary stability, even in low bone quality [18]. Thread macro-geometry also plays an important role in achieving proper primary stability [19]: Implants with deeper threads, small pitch, and reduced helix angle were shown to enhance primary stability by achieving higher bone to implant contact while reducing osseo-compression [19,20]. 

In contrast with primary mechanical stability, implant secondary stability is a dynamic biological phase characterized by bone apposition on implant surface [21]. 

This process is mainly determined by implant surface microtopography [6]. Implants with machined surfaces were first introduced, showing very good long-term results [22]. Nevertheless, implants presenting micro-rough surfaces presented increased survival rates when compared to turned surfaces and appeared to promote faster and greater bone apposition around the fixture [23,24]. Lately, nanostructured surfaces were introduced in the attempt to further improve implant to bone interplay on a cellular level by creating bioactive surfaces capable of interacting with binding proteins and osteoblasts [25]. Such surfaces showed a better bone-to-implant contact when compared to micro-rough surfaces [26].

Bone apposition on implant surface determines implant secondary stability and can be followed up by measuring ISQ values [10]. The classical scheme of implant stability is characterized by an initial loss of mechanical stability three to four weeks post-operatively, followed by a subsequent increase due to bone biological response [5]. Implant stability measurements can predict loading protocols [27]. ISQ at baseline can therefore guide immediate loading [28] and ISQ variations over the first six weeks of healing can be predictive for early loading [29]. 

Conventional loading protocols were classically performed after a two-month healing period, while early and immediate loading protocols were widely investigated in the last decade [30]. These accelerated protocols mainly relied on implants reaching high primary stability through improvements in macro-geometry along with accelerated bone apposition and maintained secondary stability by the means of surface micro-topography modifications [31]. Implant macro- and micro-geometry are thus important factors that may allow to control primary and secondary stability and guide implant future loading protocols [30].

The purpose of this clinical study is to evaluate implant stability during the first month of healing using a fixture presenting four different thread geometries that can be adapted to bone characteristics.

## 2. Materials and Methods

All patients needing implant therapy for fixed prosthetic rehabilitation were eligible for entering this study, provided that they fulfilled the following inclusion criteria:

i) Height of the residual bone crest in the programmed implant site ≥9 mm and thickness ≥7 mm;
ii) Healed bone crest (almost three months elapsed after extraction or tooth loss);
iii) Patient age > 18 years;
iv) Patients able to examine and understand the study protocol.

The following exclusion criteria were adopted:

i) Myocardial infarction within the past six months;
ii) Poorly controlled diabetes (HBA1c > 7.5%);
iii) Coagulation disorders;
iv) Radiotherapy to the head/neck district within the past two years;
v) Present or past treatment with intravenous bisphosphonates;
vi) Immunocompromised patients;
vii) Psychological or psychiatric problems;
viii) Alcohol or drug abuse;
ix) Poor oral hygiene and motivation (full mouth plaque score > 30% and/or full mouth bleeding score > 20%);
x) Uncontrolled periodontal disease.

All procedures were performed in accordance with the recommendations of the Declaration of Helsinki for investigations with human subjects, as revised in Fortaleza (2013). All patients were thoroughly informed about the procedures and signed an informed consent form. The study was approved by the Ethics Committee at the Saint Joseph University of Beirut, Lebanon (#CE346).

Preoperative evaluation included clinical examination of the edentulous ridges and natural dentition, as well as a cone beam computed tomography (CBCT) of the relevant sector. Patients underwent a prosthodontic evaluation for treatment planning and all surgeries were performed by the same experienced surgeon (C.M.) at the Oral Surgery Department, Faculty of Dental Medicine, Saint Joseph University (Beirut, Lebanon) between June 2016 and April 2017.

### 2.1. Surgical Procedure 

Patients were asked to rinse with chlorhexidine digluconate solution (0.2%) for 1 min approximately 10 min before surgery. Under local anesthesia, a crestal incision and full-thickness flap elevation were performed. Implant site preparation was initiated following a standard drilling protocol for placement of 3.3 mm core diameter implants regardless of thread diameter (AnyRidge, MegaGen, Gyeongbuk, South Korea). The surgeon clinically assessed bone density based on tactile evaluation according to Misch classification [32] as follows:

D1: Almost all dense cortical bone similar to oak- or maple-like in hardness;
D2: Homogenous, dense bone similar to white pine in hardness;
D3: Thin porous cortical and fine trabecular bone similar to balsa wood in hardness;
D4: Little or no cortical bone, with fine trabecular bone similar to Styrofoam in hardness.

**Figure 1:**
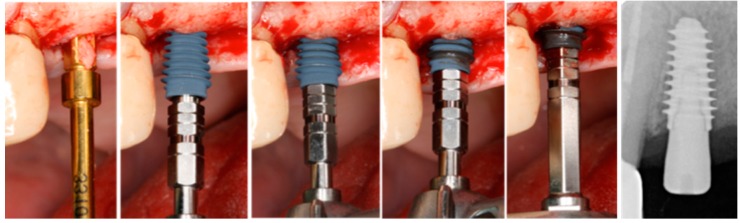
Following final 3.3 mm bone preparation, a 4.5 mm implant was inserted using an electronic torque wrench and final insertion torque was recorded.

Depending on clinical bone type assessment, the surgeon chose between 4 mm, 4.5 mm, 5 mm, or 5.5 mm diameter implants. Ten-millimeter length implants were preferentially used, and 8.5 mm length was selected when the clinical situation did not allow it. Implants were inserted using an electronic torque wrench (DTA, Studio AIP, Italy) until the final seating of implant collar at bone level and the final IT value was noted (Figure 1). An aluminum transducer (Smartpeg Type 27, Osstell, Göteborg, Sweden) was then screwed into the implant and torqued to 15 Ncm using an electronic torqueing device (Meg-Torq, MegaGen, Gyeongbuk, South Korea). A blinded operator recorded in duplicate ISQ values from mesio-distal, disto-mesial, bucco-lingual and linguo-buccal directions. Instrument calibration was verified before and after each patient visit using an implant fixed in an epoxy resin block. Smartpeg was then unscrewed and a transmucosal healing abutment was connected to the implant, and soft tissues were approximated and sutured around it for a nonsubmerged healing protocol. Periapical radiographs were then performed following paralleling long cone technique.

Patients were prescribed analgesics and antibiotic coverage (amoxicillin 2 g/daily or in case of allergy clindamycin 600 mg/daily) for 7 days, as well as oral rinses of 0.12% chlorhexidine gluconate for 15 days following implant placement. 

Patients were recalled at one, two, three, and four weeks and follow-up RFA measurement were performed for each implant using the same previously described protocol.

Impressions were taken at week three and functional provisional restorations were delivered at week four when ISQ values were > 70. Patients were then enrolled in a maintenance program and recalled every four months for periodontal and oral hygiene follow-up.

**Figure 2:**
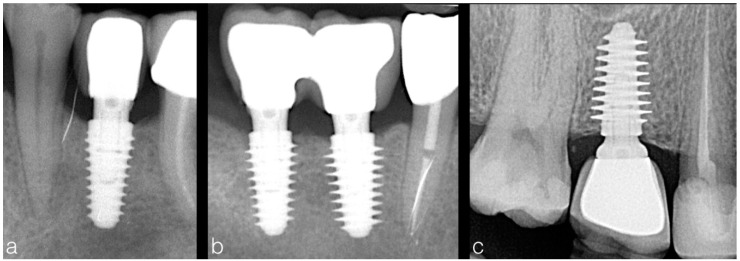
One year post-loading periapical radiographs showing implants placed in different bone density with variable thread depth (**a**—4 mm, **b**—4.5 and 5 mm, **c**—5.5 mm). Implants inserted in hard bone presented minimal thread depth (**a**) to avoid excessive bone compression, while implants with deep threads were used in soft bone (**c**) in order to attain higher primary stability.

After one year of prosthetic loading, implants were clinically evaluated and an additional periapical x-ray using paralleling long cone technique was performed (Figure 2). Marginal bone level was determined from linear measurements made on DBSWIN software (Version 5.7.1, Dürr Dental, Bietigheim-Bissingen, Germany), by two examiners (blinded to implant stability values) on each periapical radiograph from the most mesial and distal points of the implant platform to the first corresponding point of bone/implant contact, as previously described [33]. The 1:1 magnification of the x-rays was validated by measuring the known implant length and width.

### 2.2. Predictor and Outcome Variables 

This clinical prospective study tested the null hypothesis of no significant differences in IT and ISQ values between different bone types, against the alternative hypothesis of a difference. The primary predictor variable was bone density, assessed intraoperatively by the surgeon in four groups (D1, D2, D3, and D4).

Primary outcome:

- Implant stability (IT and ISQ) measured at implant insertion and during the first four weeks of healing.

Secondary outcomes:

- Marginal bone loss measured 12 months after implant insertion;
- Implant failure: Implant mobility (tested by tightening abutment screws at 35 Ncm at prosthesis delivery) or implant removal suggested by progressive marginal bone loss;
- Any complication or adverse event.

### 2.3. Statistical Analysis

Statistical analysis was performed using Statistical Package Software for Social Sciences (SPSS for Windows, version 22.0, Chicago, IL, USA). The significance level was set at 0.05.

Implant stability was described at each time interval with a single ISQ value (mean of eight measurements). Kolmogorov-Smirnov test was used to assess the normality of the continuous variables. Nonparametric tests were executed for variables not normally distributed. Kruskal-Wallis test and Friedman test for repeated measures were used to compare the continuous variables within and among the different groups of the present study. The strength of the association between IT and ISQ was assessed by Spearman Rho correlation coefficient: This evaluation was performed both for the entire sample and for each experimental group.

## 3. Results

From a total of 21 patients evaluated for entering this study, 14 consecutive patients (6 male, 8 female, mean age 52.3 ± 13.7) fulfilled all inclusion criteria and were enrolled and treated with the insertion of 40 implants (21 implants in maxilla and 19 in mandible; 33 implants with 10 mm length and 7 implants with 8.5 mm length). No dropouts occurred during the entire follow-up period of the study. Thirteen implants were inserted in D1 bone type, ten implants in D2, and D3 bone types, and seven implants in D4 bone type. 

**Figure 3:**
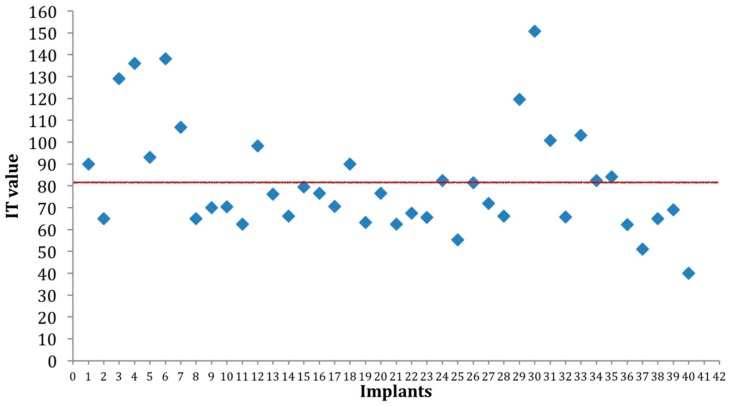
Scattered insertion torque (IT) values showed a concentration of the majority of measurements within an optimal IT value (red line), regardless of bone density. Although some higher values were recorded in hard bone, all IT values were above 40 Ncm threshold and were considered within a “comfort zone” for early or immediate loading.

**Figure 4:**
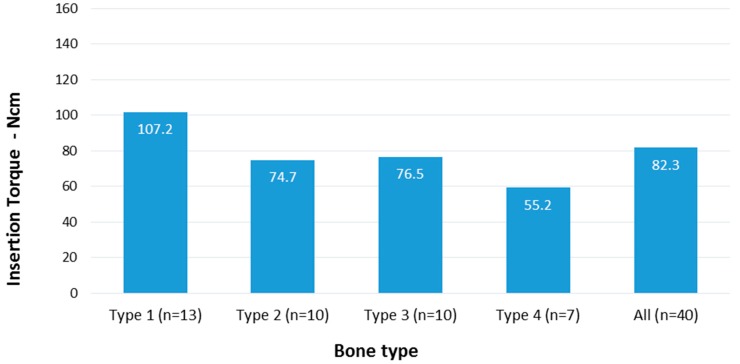
Mean IT values in different bone types. There was a significant difference between IT values in D1 and D2 bone and between D3 and D4 bone. No significant difference was noted between IT values in D2 and D3 bone.

Overall mean IT value at baseline was of 82.3 ± 33.2 Ncm. Mean IT value at baseline varied between the four different types of bone and was 107.2 ± 35.6 Ncm, 74.7 ± 14.0 Ncm, 76.5 ± 31.1 Ncm and 55.2 ± 22.6 Ncm in D1, D2, D3, and D4 bone, respectively ( Figure 3 and Figure 4). 

IT values significantly changed with bone type (p = 0.003). IT was significantly lower in D4 bone, intermediate in D2 and D3 bone (but with no significant difference between the two groups; p = 0.449), and significantly higher in D1 bone. 

No significant correlation was found between IT and implant length (r = −0.013; p = 0.937; N = 40).

**Figure 5:**
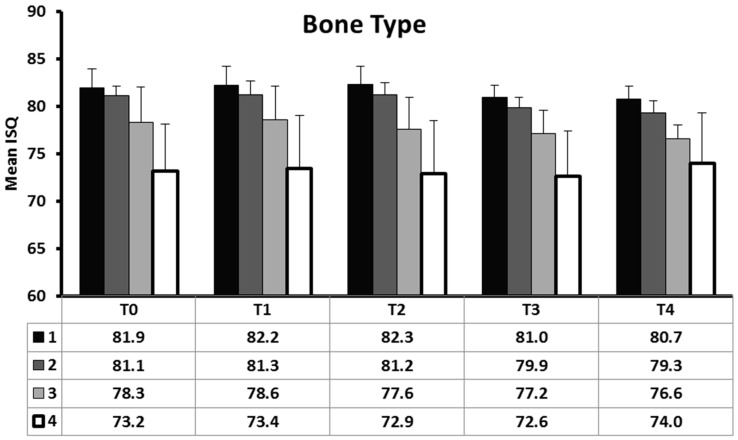
Distribution of mean ISQ at various time-points according to bone type.

Overall mean ISQ at baseline was 79.3 ± 4.3 and respective mean values in D1, D2, D3, and D4 bone qualities were 81.9 ± 2.0, 81.1 ± 1.0, 78.3 ± 3.7, and 73.2 ± 4.9. There was a significant difference in ISQ between the four different bone types at all time points (p < 0.001) (Figure 5). Regardless of measurement timing, ISQ was significantly higher in D1 and D2 bone, intermediate in D3, and lower in D4 (Table 1).

**Figure 6:**
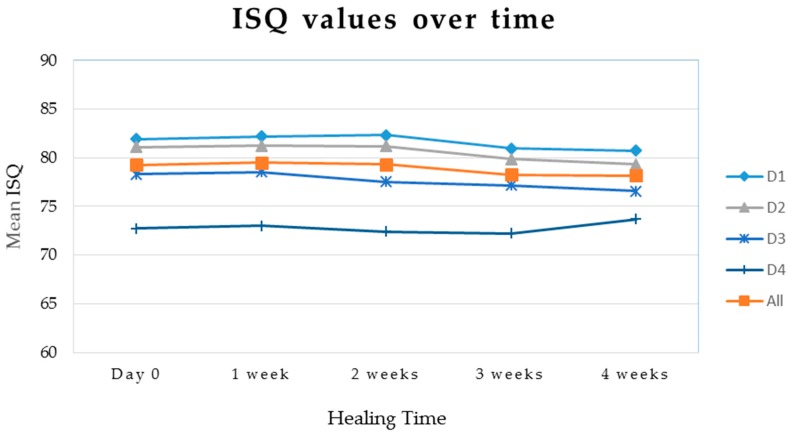
Mean ISQ values in different bone density over time. A minimal drop in ISQ values occurred at three weeks in D1, D2, and D3 classes, while no significant differences were recorded over time in D4 group (*p* = 0.07). Thirty-nine out of forty implants presented an ISQ above 70 and were considered within a “comfort zone” for early or immediate loading.

ISQ significantly changed with time when considering the entire sample (p = 0.00001). This pattern was also observed in D1 (p = 0.004), D2 (p = 0.002), and D3 (p = 0.01) groups, while ISQ variations over time in D4 bone results were not significant (p = 0.07) (Figure 6). According to Spearman Rho correlation coefficient, IT and ISQ values appear moderately associated when we consider the whole sample (r = 0.55, p = 0.0002). When we split the results into four groups according to bone density, we found nonsignificant weak direct associations between IT and ISQ in D2 (r = 0.35, p = 0.31) and D3 (r = 0.47, p = 0.16). In D1 quality, we found a nonsignificant weak inverse correlation between IT and ISQ (r = -0.32, p = 0.28). In D4 bone quality only, IT and ISQ showed a strong direct correlation (r = 0.95, p = 0.0008). In other words, in this bone quality only, an increasing IT corresponded to an increasing ISQ value. Complete results are presented in Table 2.

Four weeks after surgery, 39 out of 40 implants (97.5%) presented an ISQ value >70 and were loaded following early loading protocol, with the remaining implant being loaded after additional four weeks of healing.

Twelve months after prosthetic loading, all 40 implants had satisfactory function without the occurrence of any biological or mechanical complication. Average marginal bone loss around implants was 0.12 ± 0.12 mm, compared to baseline level (0.11 ± 0.1 on the mesial aspect, 0.13 ± 0.12 on the distal aspect). No significant correlations were demonstrated between marginal bone loss and the following variables: IT value, implant diameter, and bone type.

## 4. Discussion

Achieving high primary stability upon implant placement is essential since it helps to reduce micromotions and favor strong bone-to-implant interface, both of which are crucial for properly determining loading protocols [3,34]. 

In the present work, implants with threads of different depth were used in order to assess variable macro-geometry as a clinical way to control primary stability. These implants presented the same core diameter of 3.3 mm, but had four types of thread depth determining four different diameters of 4, 4.5, 5, and 5.5 mm. Thus, following final implant site preparation with a 3.3 mm drill, mimicking implant core diameter, and in order to avoid IT values that may impede osseointegration (very high in hard bone or very low in soft bone), it relied upon the clinician to select appropriate implant size from the four available diameters. The main determinant in this choice were therefore based on surgeon’s tactile sense and clinical judgment during drilling. Even though this represents a subjective method in the final choice of implant size, the overall result seems clinically satisfactory with optimal implant stability values setting the path for an early loading protocol. Nevertheless, a wide variability in IT and ISQ values was noted and it significantly correlated with bone density. Hence, it is very important to set comprehensive lower and upper thresholds for IT and ISQ values, in both high and soft bone density conditions, in order to define a “comfort zone” where implant osseointegration and peri-implant bone healing could be not compromised after loading.

In the present work, overall mean IT value was 82.3 Ncm, with a relatively high mean value of 55.2 Ncm in soft D4 bone, when compared to others studies with ISQ values ranging between 22 Ncm and 40 Ncm when using implants with tapered geometry [9,11]. Low implant IT was reported to favor micromotion [35], potentially jeopardizing implant osseointegration [36]. Subsequently, the choice of implants with deeper and larger thread diameter in order to obtain higher implant stability in soft bone can be considered as an effective method to enhance osseointegration at shorter healing periods, possibly affecting future loading protocols [37]. IT values above 30–35 Ncm were considered by numerous authors as the minimum threshold for immediate loading of implants [38,39]. In a previous study, it was stated that implants with IT values below 40 Ncm could not withstand a 30 Ncm abutment torqueing after six weeks of healing [10]. In the present work, no implant presented IT values below 40 Ncm, even in soft bone, hence remaining within the “comfort zone” mentioned earlier in that manuscript. 

Although IT values in D2 and D3 bone were significantly higher than in D4 bone and significantly lower than in D1, the present study showed similar IT values with no significant differences when comparing implants placed into D2 and D3 bone. Setting the difference between these two types of bone remains challenging, since it is quite difficult to accurately discern bone quality into D2 and D3 bone based uniquely on tactile perception during drilling [40]. In a previous clinical study, histomorphometric analysis of bone core biopsies retrieved during implant preparation sites could not set the difference between hand assessed D2 and D3 types of bone [9]. This difficulty in discerning between these two intermediate types of bone led to the proposal of a different bone classification dividing bone quality into Hard, Medium, and Soft bone instead of the usual four-class classification [41].

When looking into IT values in hard bone, the present results showed a mean value of 107.2 Ncm. Even though this value may be high when compared to commonly described IT values [42], it is lower than that described in some clinical studies using tapered implant geometry reporting values ranging from 120 Ncm up to 176 Ncm [9,11,43]. A recent meta-analysis concluded that high IT values do not seem to be a predictive factor for implant failure [44]. Nevertheless, in the present study, IT values reached in hard D1 quality bone could potentially introduce risks related to mechanical deformation of implant connection [45] and excessive cortical compression, potential cause of early marginal bone loss [13,46]. Marginal bone remodeling following excessive compressive forces generated by high IT on cortical bone around implant necks was described in both animal [12] and clinical studies [47]. On the other hand, other authors could not establish a relationship between high IT values and marginal bone loss around implants [43,48]. This controversy in the literature may be due, among numerous other influencing factors, to the fact that some implant geometries may distribute compressive forces better than other designs, possibly preventing marginal bone loss. In the present work, high IT did not seem to affect peri-implant bone levels, since no significant marginal bone remodeling was observed around implant necks at 12-month follow-up (mean marginal bone loss 0.12 mm). The entity of marginal bone loss is lower when compared to data commonly described in the literature, but is consistent with studies using implants with stable internal connections of good quality [49,50]. It is equally important to define an upper IT threshold for a “comfort zone” where implants can have enough primary stability to withstand early or immediate loading protocol, while avoiding detrimental peri-implant osseo-compression and mechanical issues. In the clinical reality, the use of a 3.8 mm drill before placing a 4.0 mm AnyRidge implant could be considered in the presence of hard D1 quality. This choice could help to avoid extremely high IT with possible mechanical stress to implant components while, at the same time, maintaining satisfactory implant stability. This approach is also supported by a previous study on the same implant, showing that IT > 50 Ncm subject the bone-implant system to unnecessary biological and mechanical stress without additional benefits in terms of implant stability [51].

The mean ISQ value in the present work was 79.3, with a variability mainly depending, among other factors, on IT and bone quality. When recorded in soft and hard bone, mean ISQ values showed significantly different results (73.2 and 81.9, respectively). The correlation between ISQ values and bone density was already reported in the literature with higher values in denser bone qualities [10,11]. Nevertheless, the relationship between IT and ISQ was not linear: They correlated significantly only in soft bone (D4, p = 0.0008), while the strength of their association was not significant in the other groups (D1, p = 0.28; D2, p = 0.31; D3, p = 0.16). This outcome is in accordance with a recent meta-analysis stating that IT and ISQ are independent and incomparable methods for measuring implant stability [52]. Furthermore, this result is consistent with the findings of a clinical study conducted with the same implant here tested, which demonstrated that IT and ISQ correlate significantly only when IT value was lower than 50 Ncm [51].

During the early periods of healing, a drop in ISQ values is generally described in literature at the third week. This drop is due to the bone remodeling occurring around implants during early healing phases while transitioning from initial mechanical stability to secondary biological stability [5,53]. In the present study, a slight stability drop (range: 0.9—1.2 ISQ units) was noted at the third week of healing in D1, D2, and D3 bone types, while almost no drop was noted in D4 bone (0.5 ISQ units). The slight stability drop in hard and medium quality bone may be interpreted as a greater peri-implant bone remodeling due to surgical trauma and cortical bone compression. Nevertheless, the values recorded in the present study were lower than the ones usually described in literature [10]. In a recent comparative study on two different implant thread designs, implants with knife edge threads, similar to those used in the present work, showed a negligible drop in ISQ values over time when compared to implants with V-shape threads [54]. The authors justified this finding by the fact than knife edge threads cut into the trabeculae of cancellous bone, while V-shaped threads compressed the peri-implant bone, leading to a greater remodeling process [54]. 

During the first weeks of healing, an interaction between implant surface and surrounding bone usually occurs leading to bone apposition over this surface. This phenomenon, defined as secondary stability, leads to an enhanced stability when ISQ values are measured [31]. Secondary stability is significantly influenced by implant micro-geometry and micro-rough surfaces and has been demonstrated to promote faster and greater new bone apposition in comparison to turned surfaces [53]. Implants used in the present study have a micro-rough surface characterized by the incorporation of calcium particles of nanometric scale. Nano surfaces showed an enhanced ability to bind proteins and osteoblasts, thus accelerating bone apposition on the surface [25,55,56]. Implants presenting the same surface characteristics as those used in the present work showed early bone apposition in clinical studies [57] and an enhanced bone-to-implant contact in animal histologic studies [26]. The minimal drop in ISQ values in hard and medium quality bone, along with the virtual absence of ISQ decrease in implants inserted in soft bone, can therefore be attributed not only to implant geometry but also to the surface effect leading to early bone deposition. This is especially valid in soft bone, where osteo-compression is minimal, and this biological effect can be fully expressed. 

The minimal drop in ISQ values, observed in this study at three to four weeks, may allow the application of immediate/early loading protocols even in challenging situations, such as soft bone quality. Many authors tried to set an ISQ threshold with clinical significance when planning future prosthetic loading, and values above 60–65 ISQ units were proposed for splinted immediately loaded implants [28,34], while ISQ values above 70 were considered predictive for successful early loading of partial edentulous implant cases [31]. In the present study, at the four-week loading session, 97.5% of the implants had an ISQ value above the aforementioned 70 ISQ unit threshold, identifying these implants within the “comfort zone” for an early loading protocol. 

These implants were loaded at 4 weeks and showed successful clinical outcomes at 12-month post-loading follow-up. They were placed in different bone qualities and in both mandibular and maxillary locations, showing stable results over time, irrespective of bone quality. If early loading of implants placed in the posterior mandible was already validated through clinical studies [31], loading of implants in the posterior maxilla did not reach a clinical consensus yet [30]. In the present study, both implant macro- and microgeometry were therefore crucial elements in achieving firm primary and fast secondary stability, thus keeping the implants within the “comfort zone” for early loading at four weeks after implant placement, with satisfactory short-term clinical outcomes. 

One of the main limitations of this study is represented by the subjective method used to determine bone density, which guided the clinician to the implant choice. Objective and reliable methods to evaluate and identify bone density are needed, in order to minimize possible diagnostic errors. Another limitation of this research was the small number of included subjects, together with the absence of a sample size calculation. The present results could be used to determine adequate sample size for future studies.

Furthermore, the results of the present study should not be extrapolated to all implant systems: Other geometries and/or implant site preparation techniques may lead to different clinical outcomes, and further investigations using different macro-geometries should be performed. In the future, randomized controlled studies should be performed in order to confirm the outcomes of the present clinical prospective study.

## 5. Conclusions

Bone type may affect implant primary stability by leading to higher IT values in hard bone and lower values in soft bone. Nevertheless, the present study showed that matching implant geometry to bone type may control IT values in the majority of cases leading to a “comfort zone” where optimal implant primary stability may be obtained in all bone types. Implant macro- and micro-geometry play an important role in maintaining high ISQ values during the first weeks of loading, thus allowing for successful early loading regardless of bone density. High IT values do not seem to affect marginal bone levels when using this specific implant design.

## Figures and Tables

**Table 1:** Mean IT and implant stability quotient (ISQ) in different bone types. Both IT and ISQ values resulted significantly different between groups with different bone density at each time point.

Measurement	Bone Type	Mean Value	Standard Deviation	N	*p*-value
**IT**	**All**	**82.3**	**33.2**	**40**	**0.003**
	1	107.2	35.6	13	
	2	74.7	14.0	10	
	3	76.5	31.1	10	
	4	55.2	22.6	7	
**ISQ T0**	**All**	**79.3**	**4.3**	**40**	**<0.001**
	1	81.9	2.0	13	
	2	81.1	1.0	10	
	3	78.3	3.7	10	
	4	73.2	4.9	7	
**ISQ T1**	**All**	**79.5**	**4.4**	**40**	**<0.001**
	1	82.2	2.0	13	
	2	81.3	1.4	10	
	3	78.6	3.6	10	
	4	73.4	5.6	7	
**ISQ T2**	**All**	**79.3**	**4.6**	**40**	**<0.001**
	1	82.3	1.9	13	
	2	81.2	1.3	10	
	3	77.6	3.4	10	
	4	72.9	5.6	7	
**ISQ T3**	**All**	**78.3**	**3.8**	**40**	**<0.001**
	1	81.0	1.3	13	
	2	79.9	1.1	10	
	3	77.2	2.4	10	
	4	72.6	4.8	7	
**ISQ T4**	**All**	**78.1**	**3.5**	**40**	**<0.001**
	1	80.7	1.4	13	
	2	79.3	1.2	10	
	3	76.6	1.5	10	
	4	74.0	5.3	7	

**Table 2:** Spearman Rho correlation coefficient between IT and ISQ according to the different groups.

Group	Rho Coefficient	Significance
D1	−0.32	0.28 ^NS^
D2	0.35	0.31 ^NS^
D3	0.47	0.16 ^NS^
D4	0.95	0.0008 ^S^
Overall	0.55	0.0002 ^S^

Sample = 40 (overall); 13 (D1); 10 (D2); 10 (D3); 7 (D4). Overall refers to the whole sample. ^NS^ not statistically significant correlation. ^S^ statistically significant correlation.

## References

[1] Misch C.E., Perel M.L., Wang H.-L., Sammartino G., Galindo-Moreno P., Trisi P., Steigmann M., Rebaudi A., Palti A., Pikos M.A. (2008). Implant Success, Survival, and Failure: The International Congress of Oral Implantologists (ICOI) Pisa Consensus Conference. Implant. Dent..

[2] Albrektsson T., Brånemark P.-I., Hansson H.-A., Lindström J. (1981). Osseointegrated Titanium Implants:Requirements for Ensuring a Long-Lasting, Direct Bone-to-Implant Anchorage in Man. Acta Orthop. Scand..

[3] Szmukler-Moncler S., Salama H., Reingewirtz Y., Dubruille J.H., Szmukler-Moncler S. (1998). Timing of loading and effect of micromotion on bone-dental implant interface: Review of experimental literature. J. Biomed. Mater. Res..

[4] Brunski J.B. (1999). In vivo bone response to biomechanical loading at the bone/dental-implant interface. Adv. Dent. Res..

[5] Raghavendra S., Wood M.C., Taylor T.D. (2005). Early Wound Healing Around Endosseous Implants: A Review of the Literature. Int. J. Oral Maxillofac. Implant..

[6] Davies J.E. (2003). Understanding peri-implant endosseous healing. J. Dent. Educ..

[7] Tabassum A., Meijer G.J., Wolke J.G.C., Jansen J.A. (2010). Influence of surgical technique and surface roughness on the primary stability of an implant in artificial bone with different cortical thickness: A laboratory study. Clin. Oral Implants Res..

[8] Atsumi M., Park S.-H., Wang H.-L. (2007). Methods used to assess implant stability: Current status. Int. J. Oral Maxillofac. Implants.

[9] Makary C., Rebaudi A., Mokbel N., Naaman N. (2011). Peak insertion torque correlated to histologically and clinically evaluated bone density. Implant Dent..

[10] Makary C., Rebaudi A., Sammartino G., Naaman N. (2012). Implant primary stability determined by resonance frequency analysis: Correlation with insertion torque, histologic bone volume, and torsional stability at 6 weeks. Implant Dent..

[11] Makary C., Rebaudi A., Demircioglu A., Lahoud P., Naaman N. (2017). Standard Drilling Versus Ultrasonic Implant Site Preparation: A Clinical Study at 4 Weeks After Insertion of Conical Implants. Implant Dent..

[12] Duyck J., Corpas L., Vermeiren S., Ogawa T., Quirynen M., Vandamme K., Jacobs R., Naert I. (2010). Histological, histomorphometrical, and radiological evaluation of an experimental implant design with a high insertion torque. Clin. Oral Implants Res..

[13] Marconcini S., Giammarinaro E., Toti P., Alfonsi F., Covani U., Barone A. (2018). Longitudinal analysis on the effect of insertion torque on delayed single implants: A 3-year randomized clinical study. Clin. Implant Dent. Relat. Res..

[14] Ottoni J.M.P., Oliveira Z.F.L., Mansini R., Cabral A.M. (2005). Correlation between placement torque and survival of single-tooth implants. Int. J. Oral Maxillofac. Implants.

[15] Stocchero M., Toia M., Cecchinato D., Becktor J.P., Coelho P.G., Jimbo R. (2016). Biomechanical, Biologic, and Clinical Outcomes of Undersized Implant Surgical Preparation: A Systematic Review. Int. J. Oral Maxillofac. Implants.

[16] Degidi M., Daprile G., Piattelli A. (2015). Influence of underpreparation on primary stability of implants inserted in poor quality bone sites: An in vitro study. J. Oral Maxillofac. Surg..

[17] Jimbo R., Tovar N., Anchieta R.B., Machado L.S., Marin C., Teixeira H.S., Coelho P.G. (2014). The combined effects of undersized drilling and implant macrogeometry on bone healing around dental implants: An experimental study. Int. J. Oral Maxillofac. Surg..

[18] O’Sullivan D., Sennerby L., Meredith N. (2000). Measurements comparing the initial stability of five designs of dental implants: A human cadaver study. Clin. Implant Dent. Relat. Res..

[19] Abuhussein H., Pagni G., Rebaudi A., Wang H. (2010). The effect of thread pattern upon implant osseointegration. Clin. Oral Implants Res..

[20] Lee S.-Y., Kim S.-J., An H.-W., Kim H.-S., Ha D.-G., Ryo K.-H., Park K.-B. (2015). The effect of the thread depth on the mechanical properties of the dental implant. J. Adv. Prosthodont..

[21] Berglundh T., Abrahamsson I., Lang N.P., Lindhe J. (2003). De novo alveolar bone formation adjacent to endosseous implants. Clin. Oral Implants Res..

[22] Adell R., Lekholm U., Rockler B., Brånemark P.I. (1981). A 15-year study of osseointegrated implants in the treatment of the edentulous jaw. Int. J. Oral Surg..

[23] Khang W., Feldman S., Hawley C.E., Gunsolley J. (2001). A multi-center study comparing dual acid-etched and machined-surfaced implants in various bone qualities. J. Periodontol..

[24] Wennerberg A., Albrektsson T. (2009). Effects of titanium surface topography on bone integration: A systematic review. Clin. Oral Implants Res..

[25] Mendonça G., Mendonça D.B.S., Aragão F.J.L., Cooper L.F. (2008). Advancing dental implant surface technology—From micron-to nanotopography. Biomaterials.

[26] Lee S.-Y., Yang D.-J., Yeo S., An H.-W., Ryoo K.H., Park K.-B. (2012). The cytocompatibility and osseointegration of the Ti implants with XPEED(R) surfaces. Clin. Oral Implants Res..

[27] Baltayan S., Pi-Anfruns J., Aghaloo T., Moy P.K. (2016). The Predictive Value of Resonance Frequency Analysis Measurements in the Surgical Placement and Loading of Endosseous Implants. J. Oral Maxillofac. Surg..

[28] Ostman P.-O., Hellman M., Sennerby L. (2005). Direct implant loading in the edentulous maxilla using a bone density-adapted surgical protocol and primary implant stability criteria for inclusion. Clin. Implant Dent. Relat. Res..

[29] Bornstein M.M., Hart C.N., Halbritter S.A., Morton D., Buser D. (2009). Early loading of nonsubmerged titanium implants with a chemically modified sand-blasted and acid-etched surface: 6-month results of a prospective case series study in the posterior mandible focusing on peri-implant crestal bone changes and implant stability quotient (ISQ) values. Clin. Implant Dent. Relat. Res..

[30] Gallucci G.O., Benic G.I., Eckert S.E., Papaspyridakos P., Schimmel M., Schrott A., Weber H.P. (2014). Consensus statements and clinical reco mmendations for implant loading protocols. Int. J. Oral Maxillofac. Implants.

[31] Hicklin S.P., Schneebeli E., Chappuis V., Janner S.F.M., Buser D., Brägger U. (2016). Early loading of titanium dental implants with an intra-operatively conditioned hydrophilic implant surface after 21 days of healing. Clin. Oral Implants Res..

[32] Misch C.E. (1989). Bone classification, training keys to implant success. Dent. Today.

[33] Galindo-Moreno P., León-Cano A., Ortega-Oller I., Monje A., O’Valle F., Catena A. (2015). Marginal bone loss as success criterion in implant dentistry: Beyond 2 mm. Clin. Oral Implants Res..

[34] Wentaschek S., Scheller H., Schmidtmann I., Hartmann S., Weyhrauch M., Weibrich G., Lehmann K.M. (2015). Sensitivity and Specificity of Stability Criteria for I mmediately Loaded Splinted Maxillary Implants. Clin. Implant Dent. Relat. Res..

[35] Trisi P., Perfetti G., Baldoni E., Berardi D., Colagiovanni M., Scogna G. (2009). Implant micromotion is related to peak insertion torque and bone density. Clin. Oral Implants Res..

[36] Lioubavina-Hack N., Lang N.P., Karring T. (2006). Significance of primary stability for osseointegration of dental implants. Clin. Oral Implants Res..

[37] Maiorana C., Farronato D., Pieroni S., Cicciu M., Andreoni D., Santoro F. (2015). A Four-Year Survival Rate Multicenter Prospective Clinical Study on 377 Implants: Correlations Between Implant Insertion Torque, Diameter, and Bone Quality. J. Oral Implantol..

[38] Cannizzaro G., Leone M., Ferri V., Viola P., Gelpi F., Esposito M. (2012). I mmediate loading of single implants inserted flapless with medium or high insertion torque: A 6-month follow-up of a split-mouth randomised controlled trial. Eur. J. Oral Implantol..

[39] Greenstein G., Cavallaro J. (2017). Implant Insertion Torque: Its Role in Achieving Primary Stability of Restorable Dental Implants. Compend. Contin. Educ. Dent..

[40] Trisi P., Rao W. (1999). Bone classification: Clinical-histomorphometric comparison. Clin. Oral Implants Res..

[41] Rebaudi A. (2007). The ray setting procedure: A new method for implant planning and i mmediate prosthesis delivery. Int. J. Periodontics Restor. Dent..

[42] Li H., Liang Y., Zheng Q. (2015). Meta-Analysis of Correlations Between Marginal Bone Resorption and High Insertion Torque of Dental Implants. Int. J. Oral Maxillofac. Implants.

[43] Khayat P.G., Arnal H.M., Tourbah B.I., Sennerby L. (2013). Clinical outcome of dental implants placed with high insertion torques (up to 176 Ncm). Clin. Implant Dent. Relat. Res..

[44] Berardini M., Trisi P., Sinjari B., Rutjes A.W.S., Caputi S. (2016). The Effects of High Insertion Torque Versus Low Insertion Torque on Marginal Bone Resorption and Implant Failure Rates: A Systematic Review with Meta-Analyses. Implant Dent..

[45] Teixeira A.B.V., Shimano A.C., Macedo A.P., Valente M.L.C., Reis A.C.D. (2015). Influence of torsional strength on different types of dental implant platforms. Implant Dent..

[46] Aldahlawi S., Demeter A., Irinakis T. (2018). The effect of implant placement torque on crestal bone remodeling after 1 year of loading. Clin. Cosmet. Investig. Dent..

[47] Barone A., Alfonsi F., Derchi G., Tonelli P., Toti P., Marchionni S., Covani U. (2016). The effect of insertion torque on the clinical outcome of single implants: A randomized clinical trial. Clin. Implant Dent. Relat. Res..

[48] Grandi T., Guazzi P., Samarani R., Grandi G. (2013). Clinical outcome and bone healing of implants placed with high insertion torque: 12-month results from a multicenter controlled cohort study. Int. J. Oral Maxillofac. Surg..

[49] Palacios-Garzón N., Mauri-Obradors E., Labrés X.R., Estrugo-Devesa A., Jané-Salas E., López-López J. (2018). Comparison of Marginal Bone Loss Between Implants with Internal and External Connections: A Systematic Review. Int. J. Oral Maxillofac. Implants..

[50] Tallarico M., Fiorellini J., Nakajima Y., Omori Y., Takahisa I., Canullo L. (2018). Mechanical Outcomes, Microleakage, and Marginal Accuracy at the Implant-Abutment Interface of Original versus Nonoriginal Implant Abutments: A Systematic Review of In Vitro Studies. Biomed. Res. Int..

[51] Baldi D., Lombardi T., Colombo J., Cervino G., Perinetti G., Di Lenarda R., Stacchi C. (2018). Correlation between Insertion Torque and Implant Stability Quotient in Tapered Implants with Knife-Edge Thread Design. Biomed. Res. Int..

[52] Lages F.S., Oliveira D.W.D., Costa F.O. (2018). Relationship between implant stability measurements obtained by insertion torque and resonance frequency analysis: A systematic review. Clin. Implant Dent. Relat. Res..

[53] Oates T.W., Valderrama P., Bischof M., Nedir R., Jones A., Simpson J., Toutenburg H., Cochran D.L. (2007). Enhanced implant stability with a chemically modified SLA surface: A randomized pilot study. Int. J. Oral Maxillofac. Implant..

[54] McCullough J.J., Klokkevold P.R. (2017). The effect of implant macro-thread design on implant stability in the early post-operative period: A randomized, controlled pilot study. Clin. Oral Implant. Res..

[55] Cicciù M., Fiorillo L., Herford A.S., Crimi S., Bianchi A., D’Amico C., Laino L., Cervino G. (2019). Bioactive Titanium Surfaces: Interactions of Eukaryotic and Prokaryotic Cells of Nano Devices Applied to Dental Practice. Biomedicines.

[56] Cicciù M. (2018). Nanobiomaterials in dentistry: What’s the consequent level. Eur. J. Dent..

[57] Mangano C., Shibli J.A., Pires J.T., Luongo G., Piattelli A., Iezzi G. (2017). Early Bone Formation around I mmediately Loaded Transitional Implants Inserted in the Human Posterior Maxilla: The Effects of Fixture Design and Surface. Biomed. Res. Int..

